# Elite collegiate swimmers do not meet sport nutrition recommendations during heavy training: effects of sex and within-day nutrient timing

**DOI:** 10.1080/15502783.2025.2494846

**Published:** 2025-04-18

**Authors:** Emily A. Lundstrom, Mary Jane De Souza, Keiona M. Khen, Nancy I. Williams

**Affiliations:** Pennsylvania State University, Women’s Health and Exercise Laboratory, Department of Kinesiology, University Park, PA, USA

**Keywords:** Nutrient timing, swimmers, elite athletes, macronutrient intake, within-day energy balance

## Abstract

**Background:**

Compared to the general population, athletes experience high energy expenditures requiring increased energy and macronutrient intakes to sustain training and optimize performance. While the International Olympic Committee (IOC) and International Society for Sports Nutrition (ISSN) have established recommendations for nutrient intakes, many athletes do not meet the recommended daily allowance (RDA) for the general population, and sport and sex-specific differences are not well documented. Exploration of within-day energy balance (WDEB) shows athletes may achieve energy balance by the end of the day but may present with poor WDEB. Data support that female athletes are at greater risk of nutrient deficiencies than their male counterparts, and it is unclear whether swimmers meet sport-specific nutrient intake and timing recommendations. Following our previous WDEB analysis, the purpose of this investigation was to assess dietary macronutrient intake as related to RDAs (USDA and IOC/ISSN), within-day macronutrient timing, and associated sex differences in swimmers.

**Methods:**

In elite male and female swimmers (*n* = 25; 18–22 yr), we assessed energy intake (EI), total daily energy expenditure (TDEE), macronutrient intake (fat (FAT), protein (PRO), carbohydrate (CHO)) and timing during heavy training. Frequency analysis was utilized to determine the number of athletes meeting general and athlete-specific RDAs. Repeated-measures ANOVA was used to assess nutrient timing across sex groups.

**Results:**

When compared to IOC/ISSN daily recommendations, only 6/25 swimmers met FAT intake, 7/25 met CHO intake, and 24/25 met PRO intake IOC/ISSN daily recommendations.

Males had greater EI and TDEE compared to females (*p* < 0.05). PRO consumption (% of EI) was a larger percentage of total intake in male vs females (28 ± 5% vs 23 ± 3%; F = 2.996; *p* = 0.014). No swimmers met CHO recommendations (g⋅kg^−1^) pre- or during exercise for the first daily training session. 13/25 met pre-exercise CHO recommendations, while 6/25 and 11/25 met during and post-exercise CHO recommendations for the second training session. Repeated measures ANOVA revealed effects of sex and time on intake (g⋅kg LBM^−1^⋅hr^−1^) for FAT (Sex; F = 5.659, *p* = 0.26; time; F = 12.068, *p* = 0.006) and PRO (Sex; F = 6.719, *p* = 0.016; time; F = 13.177, *p* = 0.011). There was a significant sex*time interaction for CHO consumption (F = 6.520, *p* = 0.017).

**Conclusion:**

The results from this study demonstrate significant sex-differences, indicating that most swimmers meet athlete-specific recommendations for PRO, but not CHO or FAT intake. CHO timing for pre-, during, and post-exercise was met by only 52% swimmers. Results suggest that swimmers should prioritize CHO intake, emphasized around and during training bouts.

## 1. Introduction

Athletes experience high energy expenditures requiring increased energy and macronutrient intake compared to the general population to sustain training and optimize performance [1]. Endurance athletes, such as swimmers, rely on a finely tuned combination of training, nutrition, and recovery to achieve peak performance [1]. Elite swimmers complete swim training practices and engage in other training modalities such as weightlifting and cross-training (i.e. dryland training – consisting of calisthenics, flexibility and mobility work, and other cardio-based physical activities such as high intensity interval training, running, and cycling) to complement swimming-specific training and competition. Among the crucial components of an athlete’s regimen, macronutrient intake, i.e. protein (PRO), carbohydrate (CHO), and FAT, play a pivotal role in sustaining energy, supporting physiological adaptations, and ensuring overall health [2]. Additionally, to achieve body composition and performance goals, dietary energy and macronutrient intake must reflect training and competition loads [1, 3]. More specifically, the relationship between intake and an endurance athlete’s daily diet goes beyond simple 24 hr energy intake as there is a nuanced interplay of specific nutrient requirements crucial for fueling prolonged physical exertion across the day while promoting recovery and adaptation. CHO, serving as the primary energy source, is critical for maintaining glycogen stores and providing readily available fuel during exercise [4–6]. PRO, essential for muscle repair and growth, aids in recovery and adaptation to training stress [7]. Meanwhile, FAT contributes to energy production during low-to-moderate intensity exercise and plays a role in overall health by assisting nutrient absorption, supporting the immune system and hormonal balance [8].

The Recommended Daily Allowance (RDA) was established to provide guidance on adequate levels of nutrient consumption to meet 97–98% of the healthy population’s nutrient needs [9]. However, due to high energy expenditure and macronutrient demand in athletes, RDAs do not sufficiently address the unique dietary requirements of athletes, especially during heavy training periods. Professional organizations such as the International Society of Sports Nutrition (ISSN), American College of Sports Medicine (ACSM), the International Olympic Committee (IOC), and the International Association of Athletics Federations (IAAF) [2, 10–12] have established dietary recommendations for athletes to provide guidance and ensure that athletes maintain their health and performance. However, several studies demonstrate that many collegiate athletes do not even meet the RDA recommendations for the general population, indicating that athletes need greater support to meet their nutritional needs [2]. Additionally, data support that female athletes are at greater risk of energy and nutrient deficiencies resulting from inadequate energy and macronutrient intakes to match expenditure [13–15]. Such deficiencies can compromise performance, impair recovery, negatively influence endocrine function, and lead to more serious outcomes such as the exacerbation of overtraining syndrome [16–18] and/or the development of the Athlete Triad [19, 20]. While many studies have addressed nutrient deficiencies in female and male athletes, more research is necessary to explore the effect of sex on nutrient deficiencies and the resulting health and performance outcomes.

Beyond the quantity of macronutrient intake, timing of macronutrient intake across the day can play a vital role in training adaptation and sport performance. Nutrient timing involves the intentional ingestion of various types of nutrients at specific times throughout the day to favorably impact training adaptations such as muscle strength/power, body composition, substrate utilization, and physical performance [21, 22]. Timing macronutrient consumption around training sessions significantly influences an athlete’s ability to perform during training sessions and experience beneficial physiological responses following training sessions [21, 22]. For example, pre-training meals or snacks can optimize glycogen stores and provide the necessary energy substrates for sustained performance [6]. During exercise, strategic intake of CHO can delay fatigue and enhance endurance capacity [23–25]. Post-exercise nutrition is crucial for replenishing glycogen stores [26] and initiating the muscle repair and rebuilding process [27]. Based on these findings, the ISSN has published additional recommendations regarding nutrient timing strategies [21, 22] designed to elicit specific training adaptations, such as enhancing endurance capacity, facilitating recovery, or supporting muscle hypertrophy, highlighting that the manipulation of macronutrient intake timing can potentially improve the outcomes of a training program [28, 29]. Exploration of within-day energy balance (WDEB) shows that both male and female athletes may achieve end-of-day energy balance but may present with poor WDEB, i.e. long periods throughout the day where energy intake is insufficient to meet energy demands [13, 30–34]. Poor WDEB may be associated with inadequate nutrient intake or sub-optimal macronutrient timing.

Understanding the significance of macronutrient intake and its strategic timing around and during training sessions is fundamental for optimizing an athlete’s performance and fostering proper adaptations [22]. It remains unclear whether swimmers meet sport-specific nutrient intake and timing recommendations during heavy training. Therefore, following our previous WDEB analysis [13], we aimed to assess dietary and macronutrient intake as related to RDAs (USDA and IOC/ISSN) [2, 11, 35], and macronutrient timing [21, 22] recommendations in swimmers. We hypothesized that elite male and female swimmers do not meet the recommended RDAs for PRO, CHO, and FAT as established by the IOC and ISSN during heavy training. Additionally, as we previously identified sex-based differences in within-day energy balance in this population [13], we aimed to identify if within-day macronutrient timing varied depending on sex. By testing the extent to which elite swimmers meet guidelines for current recommendations and evidence-based practices, we aim to provide insights into optimizing macronutrient consumption for the betterment of endurance athletes’ training outcomes and overall well-being during heavy training.

## 2. Materials and methods

### 2.1. Experimental design

Secondary to our within-day energy balance analysis [13], this cross-sectional study examined the eating behaviors and energy balance in 27 elite male and female Division 1 collegiate swimmers during the heaviest training phase of the NCAA swim season. The study was approved by the Pennsylvania State University Institutional Review Board (IRB#: 13613). All participants provided consent prior to study participation. Data collection occurred over 6 weeks, lasting the duration of the training phase prior to training load reduction, that occurred in preparation for championship competition. During this time, athletes are engaged in a “dry-season” protocol, where athletes completely avoid alcohol to optimize recovery from training sessions and ensure optimal performance during frequent competitions. Energy and macronutrient intake and timing, including total caloric intake, FAT, PRO, CHO were assessed utilizing the MyFitnessPal mobile application (MyFitnessPal; Under Armour Inc; Baltimore, MD). Training data were recorded, and energy expenditure data were collected from the WHOOP wearable (WHOOP Inc., Boston, MA), and laboratory-based physiological testing was performed during this period.

### 2.2. Participants

Twenty-seven members of a NCAA Division 1 Swim team in the United States participated in this study; 11 males and 16 females. All athletes were NCAA competitors, and some athletes were Olympic Trials competitors, College Swimming & Diving Coaches Association of America (CSCAA) All-Americans, and national and international team members, and competitors from varying countries. All participants were in good health, between the ages of 18–22 years and not presently injured or requiring any major training modifications.

### 2.3. Anthropometrics and body composition

Total body mass was measured using a physician’s scale (Seca Model 770; Hamburg, Germany), and height was measured by stadiometer to the nearest 0.5 cm. Body mass index was calculated as weight divided by height squared (kg/m^2^). Body composition was determined using dual X-ray absorptiometry (DXA) (Hologic Horizon-W, Model 201331) performed by an International Society of Clinical Densitometry-certified technician.

### 2.4. Training and energy expenditure

Data collection occurred during the heaviest training phase of training periodization. Weekly training routines consisted of three resistance training sessions, six in-water sessions, and one recovery session as previously described [13]. Swim training load was quantified as yds/d, yds/wk, and min/wk. Energy expended during in-pool training and resistance training sessions, and timing of expenditure bouts were measured directly using the WHOOP heart rate and 3D-accelerometry analysis (WHOOP Inc., Boston, MA) and is described as exercise energy expenditure (EEE) (kcal/d). Total daily energy expenditure (TDEE) was comprised of the sum of purposeful and non-purposeful energy expenditure and measured via WHOOP as previously described [13].

### 2.5. Daily dietary energy and macronutrient intake

Total daily energy intake (EI) was assessed continuously from 3-day diet logs utilizing Under Armour, MyFitnessPal premium mobile applications (Under Armour, Baltimore, MD) during a 72 h (3-day) collection period during data collection. MyFitnessPal has been validated for energy and macronutrient intake in the general population [36]. A study team member provided training to participants on how to use the mobile application for recording, how to scan labels of purchased food and drink as an entry for record and provided information for estimation of portion sizes for foods that were individually prepared by the participants. Participants recorded all food, beverages, or calorie-containing items consumed two weekdays and one weekend day. EI data included the time of day, and meal type (meal or snack), item, brand and method of preparation. The application provided macronutrient time-stamped data to later determine WDEB, macronutrient consumption (as g⋅d^−1^, g⋅kg^−1^⋅d^−1^, and % of total intake), EI, hourly EI, and hourly macronutrient consumption behavior. If the participants could not contemporaneously enter dietary EI data, they were instructed to adjust the timestamp to reflect the actual time of intake. EI and total daily macronutrient intake were calculated to be the composite value of all foods recorded, and reported as EI, grams and EI and grams normalized to the participant’s body weight.

### 2.6. Within-day nutrient timing

In addition to our analysis of within-day energy balance (WDEB) [13], within-day nutrient timing was quantified by examining the intake of specific macronutrients across the 24 h of the day using the MyFitnessPal dietary data. The starting point for the calculation of the WDEB and nutrition timing measurement was at midnight on the first day of food recording. Nutrient timing was assessed continuously for the 3-days assigned to participants for recording, determined as the mean macronutrient intake over the recording period. Variables were summed for each day, and the composite average of recording days was used for analysis and included: average daily dietary composition (proportion of macronutrient intake/total intake, reported as %), average daily macronutrient intake (g⋅d^−1^, g⋅kg^−1^⋅d^−1^), and average hourly macronutrient consumption (g⋅hr^−1^, g⋅kg^−1^⋅hr^−1^). This allowed for a visualization of average daily and hourly macronutrient consumption within and across a 24-h period.

### 2.7. Nutrient recommendation and timing adherence

General population RDA’s [35, 37], athlete-specific RDA’s [2, 11, 12, 38] and nutrient timing recommendations [21, 22] were used for comparative analysis. For determination of nutrient timing recommendations for pre-, during and post-training, hourly macronutrient consumption was determined before, during and after specified training times according to recommendations.

### 2.8. Statistical analysis

Data were analyzed using SPSS Statistics Software (version 26 Chicago, IL). Before analysis, each variable was tested for outliers and normality. Normality was tested using the Shapiro–Wilk statistic. Descriptive statistics, Pearson Correlations, and t-tests were performed to evaluate the relationship between macronutrient intake and variables of interest. Frequency analysis was utilized to determine the number of participants meeting general and athlete-specific RDAs. Participants were grouped for analysis by sex, and repeated-measures ANOVA with a Bonferroni post-hoc test was run and used to assess nutrient timing across on groups based on sex.

## 3. Results

### 3.1. Participants

Twenty-seven swimmers enrolled in the study. Of the 27, no swimmers dropped out, one swimmer was excluded for having incomplete data, and one swimmer was removed from analysis due to entering the lower volume training phase (“taper”) prior to the completion of data collection. Twenty-five swimmers were used for analysis. Swimmers were primarily Caucasian, with two swimmers identifying as African American/Caribbean (*n* = 1), and Latin American (*n* = 1), respectively. All athletes had access to a fueling station containing a variety of drinks and snacks at training sessions. No swimmers reported following specific diets (vegetarian, vegan, etc.) nor any known food allergies. Swimmer characteristic information is displayed in Table 1. Males exhibited greater height (*p* < 0.001), weight (*p* < 0.001), BMI (*p* = 0.03), and FFM (*p* < 0.001) compared to females. Males had significantly lower fat mass (*p* < 0.001) and body fat percentage (*p* < 0.001) compared to females.

**Table 1.** Swimmer demographics, training, energy, and macronutrient variables.

	All (n = 25)		Male (n = 10)		Female (n = 15)		
	*Mean ± SD*	*Range*	*Mean ± SD*	*Range*	*Mean ± SD*	*Range*	*p value*
**Demographics**							
Age (yrs)	20 ± 2	18–21	20 ± 1	19–21	19 ± 1	18–20	0.057
Height (cm)	179 ± 8	163–194	186 ± 5	182–191	174 ± 5	169–179	<0.001
Weight (kg)	74.2 ± 10.5	60.3–99.4	83.8 ± 8.6	75.2–92.4	67.8 ± 5.7	62.1–73.5	<0.001
BMI (kg/m^2^)	23.1 ± 1.9	19.6–26.4	24.1 ± 1.9	22.2–26.0	22.5 ± 1.9	20.6–24.4	0.063
Body Fat (%)	22.6 ± 5.3	14.2–29.5	16.6 ± 1.9	14.7–18.5	26.7 ± 1.9	24.8–28.6	<0.001
Fat Mass (kg)	16.0 ± 2.9	11.2–21.1	13.7 ± 2.1	11.6–15.8	17.7 ± 2.1	15.6–19.8	<0.001
LBM (kg)	54.0 ± 10.8	39.7–74.7	65.6 ± 6.4	59.2–72.0	46.3 ± 4.0	42.3–50.3	<0.001
FFM (kg)	56.7 ± 11.2	42.0–77.8	68.6 ± 6.7	61.9–75.3	48.7 ± 4.3	44.4–53.0	<0.001
**Training**							
Volume (yd/wk)	38569 ± 3703	34075–45250	39178 ± 4782	34396–43960	38163 ± 2892	35271–41055	0.514
Duration (min/wk)	1100 ± 41	968–1135	1079 ± 58	1021–1137	1114 ± 14	1100–1128	0.099
**Energy**							
EI avg (kcal⋅d^−1^)	2940 ± 1011	1623–5174	3959 ± 716	3243–4675	2261 ± 431	1830–2692	<0.001
EI (kcal⋅kg^−1^⋅d^−1^)	39.1 ± 10.1	21.6–63.2	47.2 ± 7.4	39.8–54.6	33.7 ± 7.7	26.0–41.4	<0.001
TDEE (kcal⋅d^−1^)	2624 ± 552	1812–3667	3185 ± 275	2910–3460	2251 ± 316	1935–2567	<0.001
EEE avg (kcal⋅d^−1^)	715 ± 265	366–1284	901 ± 279	622–1180	591 ± 171	420–762	0.002
**Macronutrients**							
CHO (g⋅d^−1^)	351 ± 115	177–597	448 ± 94	301–597	286 ± 77	177–405	<0.001
FAT (gg⋅d^−1^)	104 ± 36	48–174	140 ± 24	103–174	79 ± 17	48–112	<0.001
PRO (g⋅d^−1^)	158 ± 73	64–371	221 ± 74	138–371	115 ± 28	64–160	<0.001
CHO avg (%)	57.0 ± 6.2	42.4–66.1	54.5 ± 4.9	49.6–59.4	58.7 ± 6.5	52.2–65.2	0.096
FAT avg (%)	17.0 ± 2.8	11.7–21.4	17.6 ± 2.7	14.9–20.3	16.5 ± 2.9	13.6–19.4	0.378
PRO avg (%)	25.0 ± 5.0	17.8–37	27.9 ± 5.5	22.4–33.4	23.1 ± 3.6	19.5–26.7	0.014
CHO (g⋅kg^−1^⋅d^−1^)	4.7 ± 1.5	2.2–7.2	5.3 ± 1.1	4.1–7.2	4.3 ± 1.2	2.2–6.3	0.034
FAT (g⋅kg^−1^⋅d^−1^)	1.4 ± 0.4	0.6–2.2	1.6 ± 0.2	1.4–2.1	1.2 ± 0.3	0.6–1.7	<0.001
PRO (g⋅kg^−1^⋅d^−1^)	2.1 ± 0.9	0.8–4.1	2.6 ± 0.8	1.6–4.1	1.7 ± 0.4	0.8–2.4	<0.001

BMI = body mass index, LBM = lean body mass, FFM = fat-free mass, EI = energy intake, TDEE = total daily energy expenditure, EEE avg = average exercise energy expenditure, CHO = dietary carbohydrate intake, FAT = dietary fat intake, PRO = dietary protein intake. Bolded p-values represent significant differences determined with independent t-tests between male and female swimmer measurements. *p* < 0.05 indicates significance.

### 3.2. Training and energy expenditure

Table 1 describes the weekly and daily training volume, duration, perceived exertion, and energy expenditure for the swimmers. Each week, swimmers trained 6 days, and had one total day of rest. Training sessions during the week included: 2 h in-water training sessions, and 1-h resistance training sessions. Each week, swimmers averaged 38,850 yards (6,700 yards per training day), and trained approximately 18 h. There were no significant differences between males and females in training parameters except for EEE (kcal/day) which was greater for males than females (*p* = 0.003). TDEE was also significantly greater in males compared to females (*p* < 0.05).

### 3.3. Macronutrient intake in all swimmers

**Figure 1.**
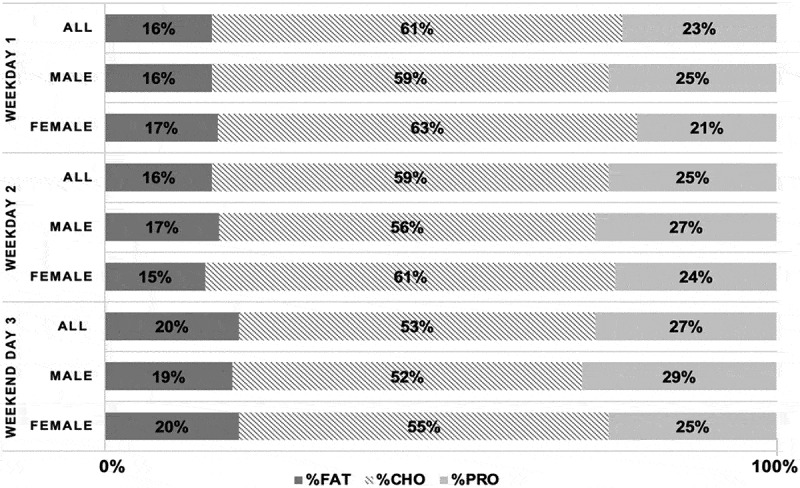
Daily distribution of macronutrient intake of all swimmers. Descriptive data of daily consumption of macronutrients are depicted for all swimmers, male and female swimmers. Distributions are presented as percentage of total daily intake, where total daily intake represents 100%. Percentages represent dietary macronutrient composition for two weekdays and one weekend day.

Energy and macronutrient intake data for all swimmers are presented in Table 1. Swimmers averaged an EI of 2940 ± 1011 kcal⋅d^−1^, and a normalized EI of 39.1 ± 10.1 kcal⋅kg^−1^⋅d^−1^. All swimmers consumed on average 351 ± 115 g⋅d^−1^ and 4.7 ± 1.5 g⋅kg^−1^⋅d^−1^ CHO; 104 ± 36 g⋅d^−1^ and 1.4 ± 0.4 g⋅kg^−1^⋅d^−1^ FAT; 158 ± 73 g⋅d^−1^ and 2.1 ± 0.9 g⋅kg^−1^⋅d^−1^ PRO. The macronutrient breakdown was 57.0 ± 5.2% CHO, 17.0 ± 2.8% FAT, 26.0 ± 5.0% PRO. Figure 1 depicts a visualization of the daily dietary composition and macronutrient distribution across the observation period for all, male and female swimmers. In all swimmers, FAT intake was a greater proportion of overall intake on weekend days compared to weekdays (20% vs. 16%; *p* < 0.05). CHO was a smaller proportion of overall intake on weekend days vs. weekdays (53% vs. 60%; *p* < 0.05). In all swimmers, PRO was a greater proportion of overall intake on weekend days vs. weekdays (27% vs. 24%; *p* < 0.05).

### 3.4. Macronutrient intake in all swimmers compared to published recommendations

Table 2 depicts the published macronutrient intake recommendations and the number and percentage of swimmers that met recommendations. Regarding general population RDA’s, 6/25 swimmers (24%) met FAT intake recommendation, while 25/25 (100%) swimmers met both the CHO and PRO recommendations. Regarding nutrient intake recommendations for athletes, only 6/25 swimmers (24%) met FAT intake, 7/25 swimmers (28%) met CHO intake, and 24/25 swimmers (96%) met PRO intake IOC/ISSN daily recommendations [2, 21, 22].

**Table 2.** Actual intake vs. Recommended intake for macronutrient consumption and nutrient timing in all swimmers.

Population	Recommendation	“n” Meeting Recommendation, (%)
**General – Daily**		
CHO (g) CHO (%)	>130 g⋅d^−1^ 45–65% total dietary intake	25/25, (100%)
FAT (%)	20–35% total dietary intake	6/25, (24%)
PRO (g) PRO (%)	46–56 g⋅d^−1^ 10–35% total dietary intake	25/25, (100%)
**Athlete – Daily**		
CHO	8–12 g⋅kg^−1^⋅d^−1^	7/25, (28%)
FAT	20% of dietary intake	6/25, (24%)
PRO	1.5–2 g⋅kg^−1^⋅d^−1^	24/25, (96%)
**Athlete – Nutrient Timing**		
***Pre Exercise***		
CHO^S1^	1–2 g⋅kg^−1^⋅d^−1^ (within 3-4hrs prior)	0/25, (0%)
CHO^S2^		13/25, (52%)
PRO^S1^	0.15–0.25 g⋅kg^−1^⋅d^−1^ (within 3-4hrs prior)	0/25, (0%)
PRO^S2^		23/25, (92%)
***During Exercise***		
CHO^S1^	30–60 g⋅hr^−1^ (within sessions >60 mins)	0/25, (0%)
CHO^S2^		6/25, (24%)
***Post Exercise***		
CHO^S1^	1–1.2 g⋅kg^−1^⋅hr^−1^ (within each hr of the 4hrs post exercise)	3/25, (12%)
CHO^S2^		11/25, (44%)
***Outside of Exercise***		
***(Meal Specific Intake)***		
PRO per meal (normalized)	0.3–0.5 g⋅kg^−1^/meal 0.3–0.5 g⋅kg^−1^/meal 0.3–0.5 g⋅kg^−1^/meal	B − 22/25, (88%) L − 17/25, (68%) D − 25/25, (100%)
PRO per meal (absolute)	15–25 g/meal	B − 21/25, (84%) L − 15/25, (60%) D − 25/25, (100%)

Frequency analysis of participant intake is compared to both general population macronutrient intake and athlete-specific macronutrient intake recommendations. CHO = carbohydrate, g = grams, PRO = protein, g/kg bw = grams per kilogram body weight, S1 = first daily training session, S2 = second daily training session. Daily recommendations for the general population sourced from NIH dietary reference intakes of macronutrients [9] and USDA dietary reference intakes of macronutrients [10]. Recommendations for athletes sourced from Thomas et al. 2016 [1, 22, 5, 21, 8], IOC consensus statement on sports nutrition 2010 [2].

### 3.5. Macronutrient timing in all swimmers compared to published recommendations

Table 2 depicts the published macronutrient intake timing recommendations and the number of athletes that met the recommendations. No swimmers (0/25) met CHO recommendations pre- or during exercise for the first daily training session. Only 3/25 swimmers (12%) met CHO recommendations for post-exercise. For the second training session of the day, 13/25 (52%) met pre-exercise CHO recommendations, while 6/25 (24%) and 11/25 (28%) met during and post-exercise recommendations. Meal-specific intake recommendations of PRO for athletes were met by the majority of swimmers. More specifically, the PRO consumption per meal (normalized to body weight) was met by 22/25 (88%) swimmers during breakfast, 17/25 (68%) swimmers during lunch, and 25/25 (100%) of swimmers during dinner. Similarly, the absolute PRO consumption per meal was met by 21/25 (84%) swimmers during breakfast, 15/25 (60%) swimmers during lunch, and 25/25 (100%) of swimmers during dinner.

### 3.6. Sex differences in energy and macronutrient intake

Sex differences in energy, macronutrient intake are presented in Table 1. Males had greater EI compared to females (*p* < 0.05). Males presented with a significantly greater intake of all macronutrients in grams (CHO: 448 vs. 286 g; FAT: 140 vs 79 g; PRO: 221 vs. 115 g) and normalized for body weight (CHO: 5.3 vs. 4.3 g⋅kg^−1^⋅d^−1^; FAT: 1.6 vs 1.2 g⋅kg^−1^⋅d^−1^; PRO: 2.6 vs. 1.7 g⋅kg^−1^⋅d^−1^) compared to females. PRO consumption was a larger percentage of average total EI in male vs females (28 ± 5% vs 23 ± 3%; *p* = 0.014). Comparing dietary composition as percentages of macronutrient intake (Figure 1) by sex, there was an effect of time for CHO and FAT intake (*p* < 0.05) whereby all swimmers consumed a diet with a larger proportion of CHO (males: 57 vs. 52%; females: 62 vs. 55%) and less FAT (males: 16 vs. 19%; females: 16 vs. 20%) during the weekdays vs weekend days, but there was no effect of sex or sex*time. Regarding percentage of diet consisting of PRO, there was an effect of time (*p* < 0.05), and sex (*p* < 0.05), whereby all swimmers consumed a diet with a smaller proportion of PRO during weekdays vs weekend days (males: 26 vs. 29%; females: 22 vs. 25%), but male swimmers had a larger percentage of PRO vs. female swimmers in their diet overall.

**Figure 2.**
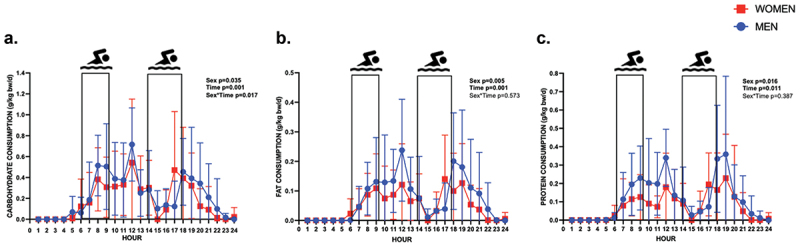
Macronutrient consumption in all swimmers across the Day. Daily consumption of macronutrients are depicted in red (women) and in blue (men). Data are presented as consumption of specified macronutrient normalized to body weight as grams/kilogram body weight/day. Black boxes indicate swim training sessions. p-values <0.05 were considered significant and denoted in bold. Panel a) CHO consumption across the day. Panel b) FAT consumption across the day. Panel c) PRO consumption across the day. Repeated measures ANOVA revealed a significant effect of sex and time on consumption of macronutrients as grams/day (g⋅d^−1)^ for all macronutrients (CHO, FAT, and PRO), (*p* < 0.05). There was a significant effect of sex and time on macronutrient intake normalized for body weight as grams/kilogram body weight/day (g⋅kg^−1^⋅d^−1^) for all macronutrients (CHO, FAT, PRO), (*p* < 0.05), and there was a significant sex*time interaction for CHO intake normalized for body weight (*p* < 0.05). g, grams; g/kg/bw/d, grams/kilogram body weight/day.

There were significant differences in macronutrient intake timing between men and women (Figure 2). Repeated measures ANOVA revealed the effects of sex and time on intake (g⋅kg^−1^⋅hr^−1^) for CHO (Sex; *p* = 0.035; time; *p* = 0.001). There was a significant sex*time interaction for CHO consumption (*p* = 0.017). Post-hoc testing revealed that there were significantly different intakes of CHO between men and women at 1500 h and 1700 h (*p* < 0.05) such that men consumed more CHO at 1500 h vs. women (0.124 g⋅kg^−1^⋅hr^−1^ vs. 0.001 g⋅kg^−1^⋅hr^−1^), and women consumed more CHO at 1700 h vs. men (0.123 g⋅kg^−1^⋅hr^−1^ vs. 0.471 g⋅kg^−1^⋅hr^−1^). Similarly, there was an effect of sex and time on intake (g⋅kg^−1^⋅hr^−1^) for FAT (Sex; *p* = 0.005; time; *p* = 0.001) and PRO (Sex; *p* = 0.016; time; *p* = 0.011) such that males consistently consumed more FAT and PRO vs women, and all swimmers consumed greater quantities of macronutrients in the later hours of the day, but no sex*time interactions were evident (*p* > 0.05).

## 4. Discussion

This study examined macronutrient intake and timing across the day in elite Division 1 male and female swimmers during heavy training preceding championship competition. The main findings of this study demonstrate that most swimmers meet recommendations for PRO, but not CHO or FAT intake for the general population. When considering athlete-specific recommendations, less than 30% of athletes met either CHO or FAT recommendations, while almost all swimmers (96%) met PRO recommendations. Additionally, nutrient timing analysis revealed that elite male and female swimmers are at risk for insufficient consumption of CHO during periods of heavy training, and that there are significant differences in intake of all macronutrients across a day between male and female swimmers. The underlying cause of these sex differences in macronutrient intake over the course of the day is not well studied, and further research is necessary to provide insight as to why differences exist. However, it is established that female athletes have a high prevalence of disordered eating behaviors and eating disorders generally and when compared to men [39–41]. Thus, as dietary restriction is a common symptom of disordered eating and eating disorders, it is possible that the female swimmers restricted the quantity and the composition of the foods they consumed compared to the men in the study, leading to the lower caloric intake as well as lower macronutrient intake across a day. The observed discrepancies in adherence to macronutrient recommendations, despite having access to fueling stations during practice sessions, emphasize the significance of educating the athletes and coaching staff on strategic timing and composition of meals and encouraging the implementation of these strategies of macronutrient consumption around training sessions, particularly for female swimmers.

The findings of this study expose the nuanced complexities surrounding macronutrient intake among competitive collegiate swimmers, highlighting both successes and areas for improvement in meeting recommended guidelines. Adhering to macronutrient recommendations is crucial for recovery from training and optimizing athletic performance in swimming. Adequate carbohydrate intake (6–12 g⋅kg^−1^⋅d^−1^) is essential for maintaining glycogen stores [42], which directly impacts endurance capacity and performance [11]. Protein intake (1.2–1.6 g⋅kg^−1^⋅d^−1^) supports muscle recovery [27], adaptation [43], and maintenance of lean body mass [44], which contribute to muscular strength and performance. Adequate fat intake (20% of total caloric intake) supports energy needs during sport performance and recovery process after events. As a calorically dense macronutrient, fat is particularly important for athletes with high energy expenditure, allowing them to meet energy demands without consuming excessive food volume [45]. Additionally, fat is essential for maintaining cell membrane structure, regulating hormones, supporting brain health, and aiding in the absorption of fat-soluble vitamins (A, D, E, and K) [46]. Its anti-inflammatory properties further contribute to reducing exercise-induced inflammation, promoting muscle recovery and overall health [47].

The adherence to the recommended daily macronutrient intake for PRO aligns with and/or exceeds IOC/ISSN [2, 21, 22] guidelines in the majority of the athletes, perhaps indicating a general awareness or deliberate effort toward meeting PRO requirements. This is important because the new ISSN position stand states that PRO requirements are higher for athletes during heavy training for maintenance of muscle mass [27]. Additionally, a higher PRO consumption can increase muscle protein synthesis and support muscle recovery following exhaustive exercise [7, 27] as the post-exercise period is characterized by increased muscle damage and protein breakdown [28].

The swimmers in our study demonstrated deficits in meeting FAT and CHO recommendations, particularly in relation to exercise sessions. This finding is consistent with other athletic populations [48,49]. One explanation for these less-than-optimal daily intakes during both weekdays and weekend days may be due to a lack of resources available to meet increased macronutrient needs, such as: food insecurity [50] a lack of food/snacks available and accessible across an entire day, time constraints, and limited or no access to nutrition monitoring [51]. Student athletes have demanding sport training schedules and busy personal lives [52, 53] that can prevent them from finding enough time to eat or eating at an optimal time. Depending on the sport, teams may have limited or no access to a sport nutrition team [54, 55], and/or have inadequate understanding of basic sports nutrition concepts for refueling [56], including lack of knowledge of the benefits of dietary CHO on training and recovery from training [56]. While FAT intake recommendations are relatively stable, adequate intake of CHO varies with the duration and intensity of exercise activity. For example, training days that involve only light exertional practices of relatively short duration, considerably less CHO is required to restore muscle and liver glycogen compared to heavier training days. Therefore, CHO intake in athletes should vary to reflect the daily training load [12]. However, during exercise at intensities greater than approximately 60% maximal oxygen consumption (VO_2_max), occurring often during phases of heavy training, blood glucose and muscle glycogen are the primary fuels oxidized to produce the ATP required to sustain exercise, increasing the CHO fuel demand [57, 58]. Taken together, these findings support our results, demonstrating noteworthy concerns regarding the optimization of general fueling strategies of endurance athletes during heavy training.

Interestingly, only a minority of swimmers managed to meet the recommended FAT intake, even with a larger proportion of intake consisting of FAT exhibited on weekend days vs weekdays. The implications of these deficits are multifaceted. FAT intake, despite often being overlooked, serves as an important energy substrate, especially during prolonged exercise bouts. A potential explanation for the low FAT intake could be due to frequent PRO supplementation practices exhibited in these athletes, which may have reduced overall whole food intake, a good source of dietary FAT, in favor of more PRO drinks or powders and thus cause a lower FAT intake.

Similarly, the shortfall in meeting CHO recommendations, especially around exercise sessions, is concerning. In this cohort of swimmers, 0% of athletes met pre- and during exercise CHO recommendations for the first training session of the day. CHO availability before, during, and after exercise is critical for sustaining performance, replenishing glycogen stores, maintaining hydration, and facilitating recovery [21, 22]. It is well established that endurance exercise performance is significantly improved when CHO is ingested during exercise [23–25, 59], and CHO during exercise reportedly maintains blood glucose levels and delays the onset of fatigue [23, 25]. Despite this, endurance athletes may utilize training strategies such as a ‘training low’ method, where athletes train in a CHO depleted state in an attempt to increase fat oxidation rates with the potential to improve endurance performance [60]. However, while research has validated that training in a CHO depleted state does in fact increase fat oxidation during exercise [61, 62], it remains unclear whether this approach is suitable for long-term training. Low CHO availability can impair high intensity exercise performance [61, 63] reducing training capacity [64], which may eventually impact overall performance, particularly in high-intensity events. As well, while fat oxidation may increase under this condition, the ability to maintain high-intensity exercise above the lactate threshold appears to be compromised in a CHO depleted state compared to undertaking exercise complement to higher CHO consumption [63]. One explanation for this inadequate CHO consumption, particularly for the first training session of the day, may be due to early training times, and potential GI discomfort accrued from consumption of a large meal prior to the training session [65, 66]. Collegiate swimmers often begin their first daily training session at 0600 h, and may not experience feelings of hunger during the early hours of the day due to their intrinsic circadian clock [67]. The low compliance observed in meeting these requirements highlights the need for targeted nutritional education and intervention strategies to optimize CHO intake strategies, aligning them with the demands of training sessions. Inadequate pre-, during, and post-exercise CHO intake during the first daily training session and deficits in meeting recommendations during the subsequent second training session indicate a potential gap in nutritional strategies surrounding training. One strategy recommended by the IOC and other groups is to increase CHO intake by means of liquid beverages or easy to consume snacks such as gels [3, 68]. Additionally, shifting back training times a few hours may not only serve to optimize sleep [62] but may also serve to give athletes more time to increase macronutrient intake and optimize pre-training fueling. Optimizing macronutrient intake timing can significantly influence glycogen replenishment, energy levels, and training adaptations, ultimately impacting an athlete’s performance and recovery [4, 25, 29, 57, 69, 70].

Sex-based differences in macronutrient consumption and overall caloric intake among male and female swimmers underscore the importance of considering individualized nutritional strategies tailored to specific athlete profiles. Male swimmers exhibited higher total caloric and intake of all macronutrients and had a greater percentage of PRO intake relative to total intake compared to their female counterparts. Additionally, male swimmers consumed more CHO before and after the second training session of the day, specifically at 1500 h. Time is has been noted to be the largest barrier to eating healthy in athletes [71]. Eating food during this time frame could be due to just convenience of the athletes having a long enough time to consume adequate amounts of food, as the bulk of classes are held until 1500 h and 1700 h. Another explanation could be that in addition to this being a large enough window for athletes to eat, the timing being such that right after the second training, athletes could eat together or among people they are familiar with which encourages increased energy intake [72]. Due to male swimmers consuming more macronutrients across the day, they may be experiencing a better overall energy status and better muscle glycogen replenishment following and preceding swim training. These differences emphasize the necessity for personalized dietary approaches, accounting for individual energy demands and sex-specific considerations.

An important overall takeaway from this study is that despite the presence of fueling stations offering snacks and drinks during training sessions, our study revealed a notable deficiency among swimmers, specifically in meeting recommended intake levels of FAT and CHO, and inadequate CHO periodization in and around training sessions. These findings demonstrate the complexity of nutritional adherence during exercise sessions, suggesting potential gaps in athletes’ understanding or implementation of dietary guidelines independent of access to fueling stations in and around practice times. It is important to consider that athletes may not have had access to these fueling stations or additional snacks during periods when they were attending university classes or fulfilling academic obligations. This highlights the potential discrepancy in nutritional support between exercise sessions and other aspects of athletes’ daily routines, suggesting a need for comprehensive strategies to ensure consistent access to appropriate fueling options throughout the demands of both athletic and academic pursuits. It is also worth noting that the offerings at the fueling station may not always align with athletes’ preferences or nutritional needs, particularly during intense practice sessions. The limited selection or lack of palatable options could contribute to athletes’ difficulty in meeting FAT and CHO intake recommendations.

## 5. Limitations

There are several limitations to this study that merit consideration. First, due to this study being a cross-sectional and observational design, it prevents the establishment of causal relationships for inadequate WDEB and nutrient intakes and timings and the potential influences on these factors. In addition, the relatively small sample size may impact the generalizability of the findings to other athletes or to other training phases within a sport’s season. Regarding the specificity of macronutrient digestion, absorption and bioenergetics, it is important to note that this study did not collect data on the type and quality of PRO and CHO consumed, which is another important consideration for both acute and chronic adaptations to exercise [63–65]. The type of PRO and the quality can influence how the body responds to the PRO [63]. PRO that is high quality (i.e. has all essential amino acids) provides more bioavailable PRO that also includes the necessary amino acids for muscle protein synthesis [63]. CHO quality is generally assessed based on the influence on blood sugar concentration, or what is called the glycemic index [64, 65]. A food with a high glycemic index correlates to a food that will rapidly and dramatically increase blood sugar concentration [64, 65]. It is generally recommended that people consume low glycemic index foods as this prevents these spikes in blood sugar at mealtime, however, high glycemic index foods may be beneficial for athletes, especially those that are consuming those foods right before or during training to assist in energy [64, 65]. We acknowledge that our study focused on elite swimmers that competed in various events that may require more specific and nuanced macronutrient intake recommendations based on the primary energy systems used for those specific events, and future research could explore event-specific nutritional strategies in more detail. Finally, the focus of this study was to investigate macronutrient intake and timing and excluded measurements and analysis of micronutrient intake. Micronutrient intake is critical for the general health and performance of the athlete. Thus, these athletes may not be meeting other recommendations that were not analyzed in this study. As well, in this sample of swimmers, there was no representation of other specific diets, such as vegetarian or vegan diets, nor any dietary restrictions. While no food allergies were reported by participants, some could have food intolerances or food beliefs that limited their intake.

## 6. Conclusion

In conclusion, while the majority of swimmers met PRO intake recommendations, deficits in FAT and CHO intake, especially around training sessions, highlight the need for tailored nutritional interventions. Addressing these gaps is crucial for optimizing fueling strategies, enhancing endurance performance, promoting proper recovery, and maximizing training adaptations among endurance athletes. Macronutrient intake analyses indicated significant sex-differences, and most athletes meet recommendations for PRO, but not CHO intake. CHO timing for pre-, during, and post-exercise was met by 52% of athletes. Results suggest that swimmers should prioritize CHO intake, emphasized around and during training. As well, future research should investigate the underlying factors influencing athletes’ dietary choices and strategies, and teams should consider diversifying the range of foods, snacks, and beverages available at fueling stations to better accommodate individual preferences and optimize nutritional support during training sessions. This tailored approach may enhance athletes’ compliance with dietary guidelines and ultimately improve performance outcomes.

## Data Availability

Data will be made available upon reasonable request.
